# Recyclable Polymer-Supported *N*-Hydroxyphthalimide Catalysts for Selective Oxidation of Pullulan

**DOI:** 10.3390/ma12213585

**Published:** 2019-10-31

**Authors:** Madalina Elena Culica, Kornela Kasperczyk, Raluca Ioana Baron, Gabriela Biliuta, Ana Maria Macsim, Andrada Lazea-Stoyanova, Beata Orlinska, Sergiu Coseri

**Affiliations:** 1 “Petru Poni” Institute of Macromolecular Chemistry of Romanian Academy, 41 A, Gr. Ghica Voda Alley, Iasi 700487, Romania; culica.madalina@icmpp.ro (M.E.C.); baron.raluca@icmpp.ro (R.I.B.); biliuta.gabriela@icmpp.ro (G.B.); ana.iurascu@icmpp.ro (A.M.M.); 2 Department of Chemical Organic Technology and Petrochemistry, Silesian University of Technology, Krzywoustego 4, 44 100 Gliwice, Poland; kornela.kasperczyk@gmail.com (K.K.); Beata.Orlinska@polsl.pl (B.O.); 3 National Institute for Lasers, Plasma and Radiation Physics, 409 Atomistilor Street, Magurele 77125, Romania; andrada@infim.ro

**Keywords:** pullulan, oxidation, NHPI, *N*-Hydroxyphthalimide, polymer-supported catalysts

## Abstract

This paper proposes a convenient route to oxidize the –CH_2_–OH groups in the water-soluble pullulan, using a new catalytic polymer-supported *N*-hydroxyphthalimide (NHPI) immobilized on polystyrene. The protocol involves the presence of sodium hypochlorite and sodium bromide. The conversion is possible at room temperature, atmospheric pressure, and pH = 10. The characterization of both the catalysts and oxidized pullulan was done using NMR and FTIR methods. Using polyelectrolyte titration with end-point indication by means of a particle-charge detector (PCD), we were able to assess the degree of electrokinetic charge in all oxidized samples as a consequence of the conversion of the –CH_2_–OH group into –COOH moieties. The possibility of recovery and recycling of the polymer-supported NHPI catalyst was tested for up to four cycles, since the morphological analyses performed on the catalysts using SEM revealed no significant changes.

## 1. Introduction

**Figure 1:**
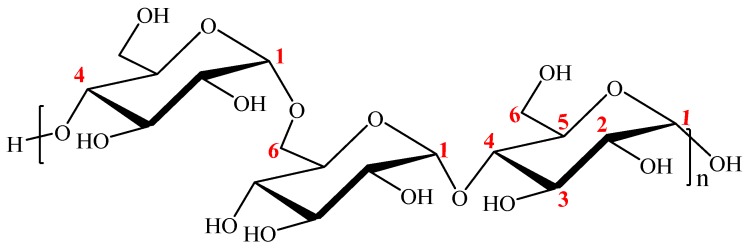
Chemical structure of pullulan.

Natural polymers to be used in different applications must often undergo chemical transformations. Among these transformations, oxidation presents a convenient approach to the alteration of the polysaccharide backbone by introduction of new functionalities, such as aldehyde or carboxyl groups, due to its relative simplicity and selectivity. Since the pioneering work of Yackel and Kenyon [1], many oxidation protocols have been reported [2,3]. In recent years, the introduction of the TEMPO (2,2,6,6-tetramethyl-1-piperidinyloxy) radical as mediator in this kind of transformation has led to a tremendous boost in polysaccharide oxidation research. However, few existing reports take into account the depolymerization side reactions which are likely to occur during longer exposure of reactants and oxidized products to the reaction medium [2,3]. As an alternative, *N*-hydroxyphthalimide (NHPI) has been proposed to replace the TEMPO catalyst in many oxidation processes [4,5,6], including in the oxidation of cellulose. In the oxidation processes of cellulose, NHPI would react with a cocatalyst in order to produce phthalimide-*N*-oxyl radical (PINO), which would initiate the reaction cycle [7]. Several procedures to generate PINO radical from NHPI have been reported, e.g., electrocatalytic, biocatalytic, and chemocatalytic systems [6]. In 2009, Coseri et al. reported that cellulose could be oxidized in the presence of NHPI and various metallic or nonmetallic cocatalysts at room temperature and in the presence of sodium hypochlorite and sodium bromide [8]. Other nonpersistent nitroxyl radicals have subsequently been proven to effectively oxidize the viscose fibers at room temperature and alkaline pH [9,10]. Pullulan is a biodegradable polysaccharide produced by the fungus *Aureobasidium pullulans*. It is composed of maltotriosyl repeating units linked via α-1,6-glycosidic bonds (Figure 1) [11]. Pullulan exhibits good biocompatibility and enzymatic degradation, making it a promising material for a wide range of applications, including pharmaceutics, food, and cosmetics [12]. Chemical functionalization is a valuable method often employed to alter the properties of pullulan, as well as to enhance its applicability in various fields.

Chemical modifications of pullulan such as oxidation [13], etherification [14], esterification [15], and hydrogenation [16] have been reported. The oxidation of pullulan to carboxypullulan is an important direction to functionalization of the hydroxyl groups in the maltotriose unit of pullulan. The introduction of carboxylic acid groups in the pullulan backbone provides anionic polymers, which are able to interact more effectively with other charged compounds. Thus, pullulan can be readily oxidized at primary hydroxyl groups to the carboxylic moiety using the free nitroxyl radical, TEMPO (2,2,6,6-tetramethyl-1-piperidinyloxy), and a primary oxidant such as sodium hypochlorite (NaOCl). In this process, the nitrosonium salt is the key species resulting from the conversion of the nitroxyl radical in the presence of NaBr/NaOCl [17]. The resulting oxidized pullulan has recently been found useful in applications for incorporation of magnetic nanoparticles used in magnetic resonance imaging [18], as well as in drug delivery, in preparation of mucoadhesive buccal films loaded with enalapril maleate [19]. According to the current trends in catalysis, attempts have been made to immobilize NHPI onto solid supports in order to obtain easily separable and reusable catalysts. For example, polymers [20], silica gel [21], and zeolite [22] were applied as solid supports. However, until now, these solid catalysts have been applied only for the aerobic oxidation of low molecular organic compounds, such as toluene, p-methoxytoluene [20,21], and 1-phenylethanol [23]. In our study, *N*-hydroxyphthalimide immobilized on polystyrenes via amide or ester bonds has been employed as a new class of catalysts for pullulan oxidation, which was chosen as a model polysaccharide due to its excellent water solubility, allowing the easiness of the work-up reaction. In this way, we expanded the use of solid catalysts based on NHPI on polysaccharides for the first time. The reactions were performed in water (the sole solvent) at room temperature, atmospheric pressure, and pH = 10. The oxidation process requires the presence of NaBr and NaOCl as cocatalysts. The possibility of recovery and recycling of the catalysts has been evaluated.

## 2. Experimental Section

### 2.1. Materials 

Pullulan (Mw = 9.98 – 10.4 × 10^4^ g/mol) was purchased from TCI Europe. Before experiments, it was dried under vacuum at 100 °C for 48 h. TEMPO (99% Aldrich), sodium hypochlorite (NaOCl, 12% chlorine, Chemical Company Romania, Iasi, Romania), sodium bromide (99% Alfa Aesar, Bucuresti, Romania), trimellitic anhydride (98% Aldrich), hydroxylamine hydrochloride (99% Acros Organic, Bucuresti, Romania), MeOH (POCH, Gliwice, Poland), and EtOH (POCH) were used without further purification. Pyridine (POCH) and trimethylamine (99% Acros Organic) were purified by distillation. Solvents: dichloromethane (DCM), 1,2-dichloroethane (DCE), *N,N*-dimethylformamide (DMF), tetrahydrofuran (THF), and 1,4-dioxane were supplied by POCH or Acros Organic and dried before use. Millipore water of HPLC grade for the dissolution of pullulan and oxidized products was used. Commercial resins (aminomethyl)polystyrene (APS-NH_2_) and Merrifield resins (chloromethyl)polystyrene (AMPS-Cl, BMPS-Cl) were supplied by Acros Organic.

### 2.2. Preparation of Catalysts

#### 2.2.1. Immobilization of NHPI by Amide Bond Using (aminomethyl) Polystyrene Resin

Synthesis of APS-NHCO-NHPI-1

First, 20 mmol of trimellitic anhydride chloride and 28 mmol of triethylamine were added at 0 °C to 1 g of commercial (aminomethyl) polystyrene polymer resin (APS-NH_2_) pre-swelled in 25 mL of DCM. The mixture was kept for 3 h at 0 °C and 24 h at room temperature, under stirring. The resulting product was filtered and washed with the following solvents: DCM, DMF, THF. After drying under vacuum, immobilized anhydride (APS-NHCO-TA) was added to 40 mL of pyridine: DCE mixture (3:1, *v*/*v*) followed by addition of 20 mmol of hydroxylamine hydrochloride. The mixture was stirred for 24 h at 75 °C. The product APS-NHCO-NHPI-1 was filtered and washed with various solvents (H_2_O, MeOH, DMF, THF, DCM, and EtOH) and then dried under vacuum. A total of 1.51 g of immobilized NHPI was obtained. Commercial resin (APS-NH_2_) and NHPI immobilized by amide bond (APS-NHCO-NHPI-1) were characterized by FTIR. Elemental analysis of APS-NH_2_, APS-NHCO-TA, and APS-NHCO-NHPI-1 was performed and used for NHPI loading calculation.

APS-NH_2_ (Acros Organics): loading 4.0 mmol NH_2_/g, microporous 200–400 mesh, 2% divinylbenzene, IR: 3310, 3060, 2895, 2820, 1670, 1616, 1454, 1450, 1442, 1360, 1320, 1230, 1190, 1150, 1060, 957, 760, 706, 695 cm^−1^; found: C 83.80%, H 7.92%, N 5.84%. 

APS-NHCO-TA: Found: C 71.84%, H 5.57%, N 2.12%.

APS-NHCO-NHPI-1: loading 1.60 mmol NHPI/g, IR: 3265, 3060, 2909, 1778, 1715, 1655, 1629, 1543, 1509, 1495, 1490, 1445, 1354, 1320, 1295, 1246, 1178, 1154, 1152, 1138, 954, 934, 904, 851, 808, 745, 701, 695 cm^−1^; found: C 75.40%, H 6.44%, N 3.75%.

#### 2.2.2. Immobilization of NHPI by Ester Bond Using (Chloromethyl) Polystyrene Resin

Synthesis of AMPS-OCO-NHPI-1

First, 22 mmol of trimellitic anhydride and 55 mmol of triethylamine were added to 1 g of Merrifield resins (chloromethyl)polystyrene (AMPS-Cl) pre-swelled in 30 mL of 1,4-dioxane. The reaction was carried out under reflux for 48 h. The resulting product was separated by filtration, and washed at 50 °C with H_2_O, MeOH, THF, and DCM. After drying under vacuum, immobilized anhydride was added to 110 mL of pyridine:DCE mixture (3:1, *v*/*v*) followed by addition of 55 mmol of hydroxylamine hydrochloride. The mixture was allowed to react for 24 h at 75 °C, under stirring. The product AMPS-OCO-NHPI-1 was filtered and washed at room temperature with H_2_O, H_2_O:MeOH (1:1, *v*/*v*) and at 50 °C with MeOH, DMF, THF, and DCM and then dried under vacuum. A total of 1.66 g of AMPS-OCO-NHPI-1 was obtained. Merrifield resin (AMPS-Cl) and NHPI immobilized by ester bond (AMPS-OCO-NHPI-1) were characterized by FTIR. Elemental analysis of AMPS-Cl and AMPS-OCO-NHPI-1 was performed and used for NHPI loading calculation.

AMPS-Cl (Acros Organics): loading 5.5 mmol Cl/g, macroporous, 16–50 mesh, 5.5% DVB, IR: 3008, 2913, 1615, 1512, 1447, 1418, 1263, 1211, 1175, 1114, 1017, 908, 825, 745, 705 cm^−1^; found: C 73.19%, H 7.29%.

AMPS-OCO-NHPI-1: loading 2.1 mmol NHPI/g, IR: 3425, 2944, 1722, 1615, 1515, 1457, 1365, 1270, 1230, 1179, 1105, 1070, 1000, 824, 748, 711 cm^−1^; found: C 68.75%, H 5.50%, N 2.89%.

Synthesis of BMPS-OCO-NHPI-2

First, 12.8 mmol of trimellitic anhydride and 32 mmol of triethylamine were added to 1 g of polystyrene (BMPS-Cl) pre-swelled in 25 mL 1,4-dioxane Merrifield resins (chloromethyl) and kept under reflux for 48 h. The resulting product was separated by filtration and washed at 50 °C with H_2_O, MeOH, THF, and DCM. After drying under vacuum, immobilized anhydride was added to 65 mL of pyridine:DCE mixture (3:1, *v*/*v*) followed by addition of 32 mmol of hydroxylamine hydrochloride. The mixture was allowed to react for 24 h at 75 °C, under stirring. The product BMPS-OCO-NHPI-2 was filtrated and washed at room temperature with H_2_O, H_2_O:MeOH (1:1, *v*/*v*) and at 50 °C with MeOH, DMF, THF, and DCM, and then dried under vacuum. A total of 1.30 g of BMPS-OCO-NHPI-2 was obtained. Merrifield resin (BMPS-Cl) and NHPI immobilized by ester bond (BMPS-OCO-NHPI-2) were characterized by FTIR. Elemental analysis of BMPS-Cl and BMPS-OCO-NHPI-2 was performed and used for NHPI loading calculation.

BMPS-Cl (Acros Organics): loading 2.8–3.2 mmol Cl/g, microporous, 200–400 mesh, 2% DVB, IR: 3020, 2912, 1604, 1508, 1494, 1450, 1417, 1267, 1115, 1107, 904, 814, 764, 705, 675 cm^−1^. Found: C 81.47%, H 7.88%.

BMPS-OCO-NHPI-2: loading 0.9 mmol NHPI/g IR: 3460, 3017, 2934, 1721, 1614, 1508, 1489, 1474, 1357, 1269, 1240, 1180, 1115, 1061, 1015, 951, 812, 749, 701 cm^−1^; found: C 73.39%, H 5.93%, N 1.28%.

The loading of NHPI in APS-NHCO-NHPI-1, AMPS-OCO-NHPI-1, and BMPS-OCO-NHPI-2 resins is slightly different in comparison with previous ones [20], as Table 1 highlights. Solid supports ((aminomethyl) polystyrene and (chloromethyl) polystyrene resins) were delivered by the same company, Acros Organics. However, it could be a different batch of product. However, the key issue regarding the NHPI loading, as has been carefully determined after each series of experiments, is to have the exact value of the incorporated NHPI. 

### 2.3. Preparation of the Oxidized Pullulan

Pullulan (1 g) was firstly dissolved in Millipore water (100 mL) at 25 °C. APS-NHCO-NHPI-1 (APS), AMPS-OCO-NHPI-1 (AMPS) or BMPS-OCO-NHPI-2 (BMPS) catalyst (0.8 g), and NaBr (0.1 g) were added to the solution of pullulan under vigorous stirring. Next, 10% sodium hypochlorite solution (10 mL) was added to the pullulan solution. The pH of the system was maintained at 10 by addition of 2 M sodium hydroxide solution and 2 M HCl solution. After 3 h, the reaction was stopped with ethanol (5 mL), the mixture was filtered off, and the recovered catalyst was extensively washed with distilled water and dried at 100 °C for 24 h as preparation for the next reaction cycle. The supernatant was precipitated using acetone, followed by centrifugation. Subsequently, the oxidized pullulan was redissolved in Millipore water, and the oligomers were removed by diafiltration through a Millipore polyethersulfone ultrafiltration membrane (cut-off: 10 kD). Diafiltration was terminated when the conductivity of the filtrate was lower than 10 µS/m, and the polymer was recovered by freeze-drying. Each catalyst was recovered and recycled for another 3 cycles of oxidation.

### 2.4. Fourier-Transform Infrared Spectroscopy (FTIR)

The FTIR spectra of the pullulan and modified pullulan were recorded using a Vertex 70 spectrometer (Bruker, Karlsruhe, Germany) with 4 cm^−1^ resolution in KBr pills. FTIR spectra of resins were recorded on a Nicolet 6700 FT-IR Spectrometer.

### 2.5. ^1^H and ^13^C NMR

All spectra were recorded in D_2_O (0.7 mL) by dissolving 10 mg of sample for ^1^H NMR, or 50 mg for ^13^C NMR. The ^1^H-NMR and ^13^C-NMR spectra were acquired using a 400 MHz spectrometer Bruker Avance DRX (Rheinstetten, Germany)., with a 5 mm QNP direct detection probe and z-gradients, using trimethylsilylpropanoic acid (TSP) as internal standard.

### 2.6. Elemental Analysis 

Elemental analyses of solid catalysts were performed on a CHNS Vario Micro Cube (Elementar Analysensysteme GmbH, Langenselbold, Germany).

### 2.7. Zeta-Potential (ζ) Measurements

A Zetasizer model Nano ZS produced by Malvern Instruments (Worcestershire, UK), was used to determine zeta potential (ζ) using a dynamic light scattering method. The instrument is equipped with a red laser 633 nm (He/Ne). Electrophoretic mobility (*μ*) measured at 25 °C was used to calculate zeta potential (ζ). When kα>>1 (*k*-Debye-Hűckel parameter and *α*-particle radius), the Smoluchowski relationship can be used, Equation (1):

(1) ς=ημ/ε

where *η* is the viscosity and *ε* the dielectric constant.

### 2.8. Polyelectrolyte Titration 

Polyelectrolyte titration with end-point indication by means of particle-charge detector (PCD 03, Mütek GmbH, Herrsching, Germany) was performed to assess the electrokinetic charge (EC) of the oxidized samples. The instrument ascertains the potential of an in situ non-stoichiometric polyelectrolyte complex formed in solution by determination of the point of zero charge (*pzc*). In order to form the complex with the oxidized pullulan sample, a volume of 10^−3^ M poly (diallyldimethylammonium chloride) solution was introduced until the *pzc* was reached, and the exact volume was used to determine the concentration of oxidized pullulan in the supernatant solution. 

### 2.9. Scanning Electron Microscopy (SEM)

The size, shape, and morphology of the polymeric microspheres before and after chemical treatment were analyzed using a scanning electron microscope (SEM), an FEI Inspect S50 apparatus (Hillsboro, OR, USA), owned by I.N.F.L.P.R. The images used in this paper are secondary electron (SE) images and were obtained due to electrons generated by a tungsten filament. The acceleration voltages and magnifications were 2–5 kV and 50–20,000x, respectively. Prior to SEM investigations, the microspheres were coated with a 5 nm Au layer by a sputtering Cressington 108 auto sputter coater for standard SEM/EDX applications, equipped with a Cressington MTM-20 thickness controller.

## 3. Results and Discussions

**Figure 2:**
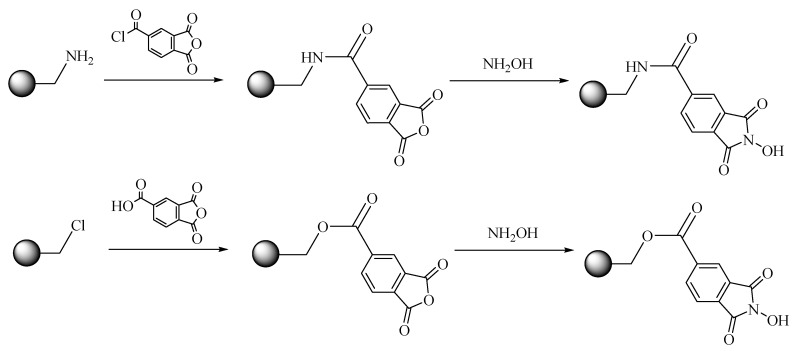
*N*-hydroxyphthalimide (NHPI) immobilization on various polymer supports via amide or ester bonds.

NHPI was successfully immobilized on polymer supports via amide or ester bonds according to the procedure reported in [20] (Figure 2). Commercial resins (aminomethyl) polystyrene and (chloromethyl)polystyrene were used as supports. They differ in loading of functional group (–NH_2_ or –Cl), crosslinking degree, and mesh size. The prepared catalysts were characterized by FTIR analysis. FTIR spectrum of APS-NHCO-NHPI-1 exhibited peaks at 3265, 1778, 1715, 1655, 1543, and 1490 cm^−1^ of *N*-hydroxyl group and carbonyl groups, as well as (O)C–N(H) and (C)N–H in amide bonds. Analogously, characteristic absorptions of *N*-hydroxyl group, carbonyl groups, and (O)C–O in ester bonds were identified at 3425, 1720, and 1232 cm^−1^ for AMPS-OCO-NHPI-1 and at 3460, 1722, and 1241 cm^−1^ for BMPS-OCO-NHPI-2. The loading of NHPI was determined based on elemental analysis.

### 3.1. Pullulan Oxidation

**Figure 3:**
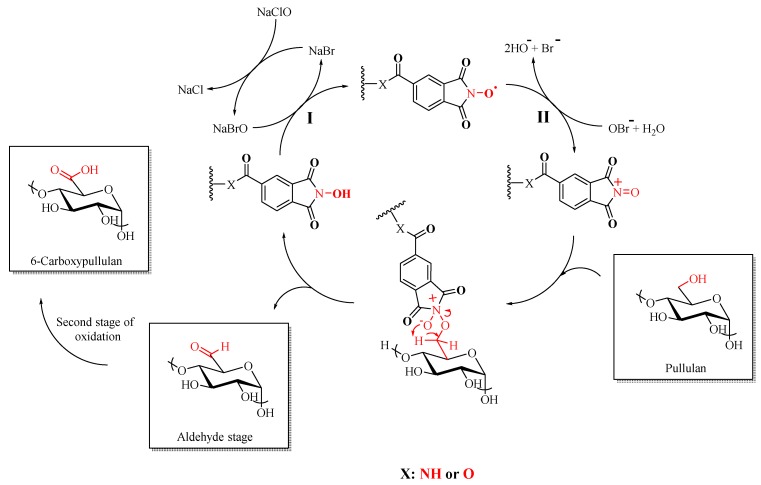
Proposed reaction mechanism for the pullulan oxidation in the presence of NHPI-supported catalysts/NaClO/NaBr.

Figure 3 presents the reaction mechanism of pullulan oxidation in the presence of the NHPI-supported solid catalysts, NaClO/NaBr. As shown in Figure 3, the hydroxyl group of pullulan at the C6 is selectively oxidized to the carboxyl group via intermediate aldehyde. Under reaction conditions, the catalyst is in situ converted into nitroxyl radical in the presence of the NaOCl/NaBr (**I**). In addition, the nitroxyl radical is converted into more powerful nitrosonium salt, the actual oxidant from system (**II**). Furthermore, the nitrosonium salt reacts with the primary hydroxyl group of pullulan and forms an aldehyde intermediate in a first stage, which is then oxidized further to the carboxyl group. After each reaction, the catalysts were recovered and reused, see Table 2. The catalytic activity of the NHPI was observed and evaluated using FTIR, NMR, and particle charge detector techniques.

### 3.2. NMR Analyses

**Figure 4:**
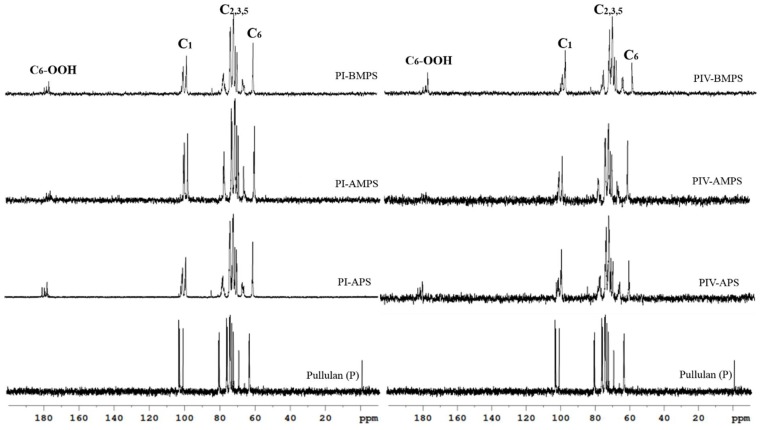
Selected ^13^C-NMR spectra of the pullulan obtained after oxidation in the presence of NHPI-supported catalysts catalysts/NaClO/NaBr.

After each oxidation cycle, the resulting products were subjected to the ^1^H-NMR and ^13^C-NMR analyses. Since ^1^H-NMR spectra for polysaccharides are difficult to discriminate due to high overlapping of the proton signals, more useful information can be acquired from ^13^C-NMR, seen in Figure 4 and Figures S1–S6 in Supplementary Materials attached to this paper. The spectra of pullulan and oxidized pullulan present characteristic peaks of the C6 atoms around 63–60 ppm. In addition, the signals of C1 atoms are around 100–104 ppm, C4 atom signals are around 8076 ppm, and a multitude of signals assigned to atoms C_2,3,5_ are located between 69 and 76 ppm [24,25,26]. After oxidation in the pullulan sample, a new peak in the region 170–180 ppm corresponding to the carboxylic acid, which was missing in the original sample, is clearly visible.

The intensity of the newer peak appearing after oxidation is linked with the number of carboxyl groups introduced during oxidation. The electrokinetic charge (EC, %) of each reaction product was determined using a particle charge detector (PCD 03). This technique measures the amount of electrokinetic surface charge directly, combining an electrokinetic probe with titration of a charge-compensating polyelectrolyte. This technique is quite simple, and no additional model assumptions are required. The results shown in Table 2 show differences in the EC of pullulan after using the three catalysts. It appears that the BMPS catalyst possesses the highest performance, followed by APS and AMPS. For each catalyst, it can be seen that its efficiency does not decrease with subsequent reuse; by contrast, a slight increase of the EC of the oxidized samples can be observed when the catalysts have been reused for the third or fourth time, especially in the cases of BMPS and APS catalysts. To test the catalysts’ efficiency, key control reactions were performed in the absence of any catalyst. Not surprisingly, as revealed by ^13^C-NMR spectra, no oxidation was observed in this case. 

### 3.3. FTIR Analyses

**Figure 5:**
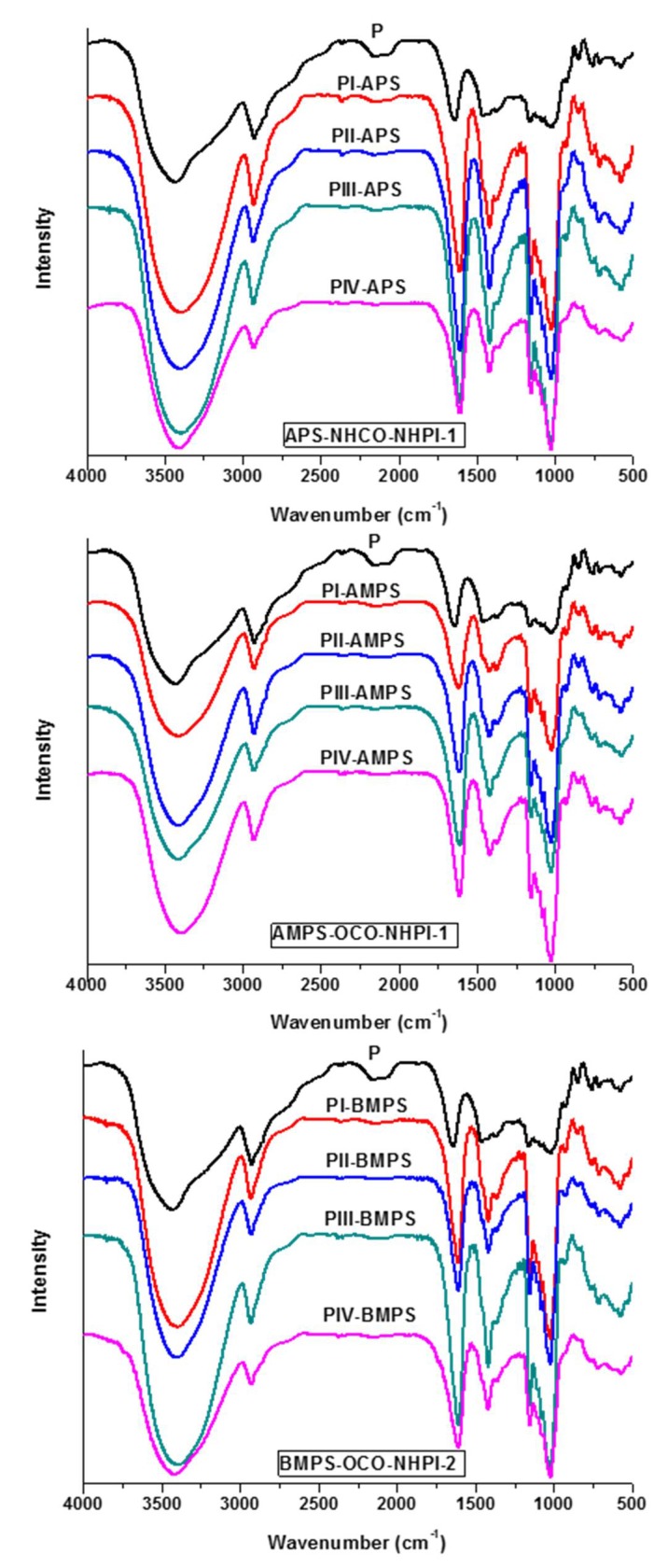
FTIR spectra of pullulan oxidized in the presence of NHPI-supported catalysts catalysts/NaClO/NaBr.

Comparison of the FTIR spectra of pullulan and oxidized pullulan in Figure 5 proves the success of oxidation, even for the case where the solid catalysts were reused three times. The infrared spectra of both pullulan and its oxidized derivative display a strong adsorption band at 3424 cm^−1^ due to the OH stretching vibration bond and an adsorption band at 2924 cm^−1^ due to the sp^3^ C–H bond. Another characteristic band of pullulan at 1638 cm^−1^ is attributed to the stretching vibration of O–C–O. The modified pullulan showed a band at 1614 cm^−1^ attributed to the C=O stretching of the free carboxylate groups, and the band at 1420 cm^−1^ is assigned to the C–O symmetric stretching of dissociated carboxyl groups. Other changes that occurred after oxidation are visible in the 1000–1300 cm^−1^ FTIR region by increasing the absorption intensity of some bands as follows: the stretching vibration of C–O at 1200‒1030 cm^‒1^, the stretching vibration of C–O–C at α-1, and 4-glycosidic linkage at 930 cm^−1^ and 1025 cm^−1^ due to the C–CO. These results indicated that the primary hydroxyl groups in pullulan were converted to carboxyl groups in the presence of the new proposed catalytic systems. Further, after oxidation, the spectra of the samples do not essentially differ. This can suggest that the catalysts are still efficient after three reutilizations, being able to perform the oxidation reaction of the primary OH groups.

The zeta potentials of all oxidized samples were measured in deionized water at a concentration of 1 mg/mL. The resulting values are strongly dependent on the number of negatively charged groups introduced after oxidation, ranging from ζ of −16.2 ± 0.4 mV (DO = 15) in the sample oxidized with AMPS, recycle 2, to ζ of −27.3 ± 0.6 mV (DO = 29) in the sample oxidized with BMPS, recycle 3.

### 3.4. SEM Analyses

**Figure 6:**
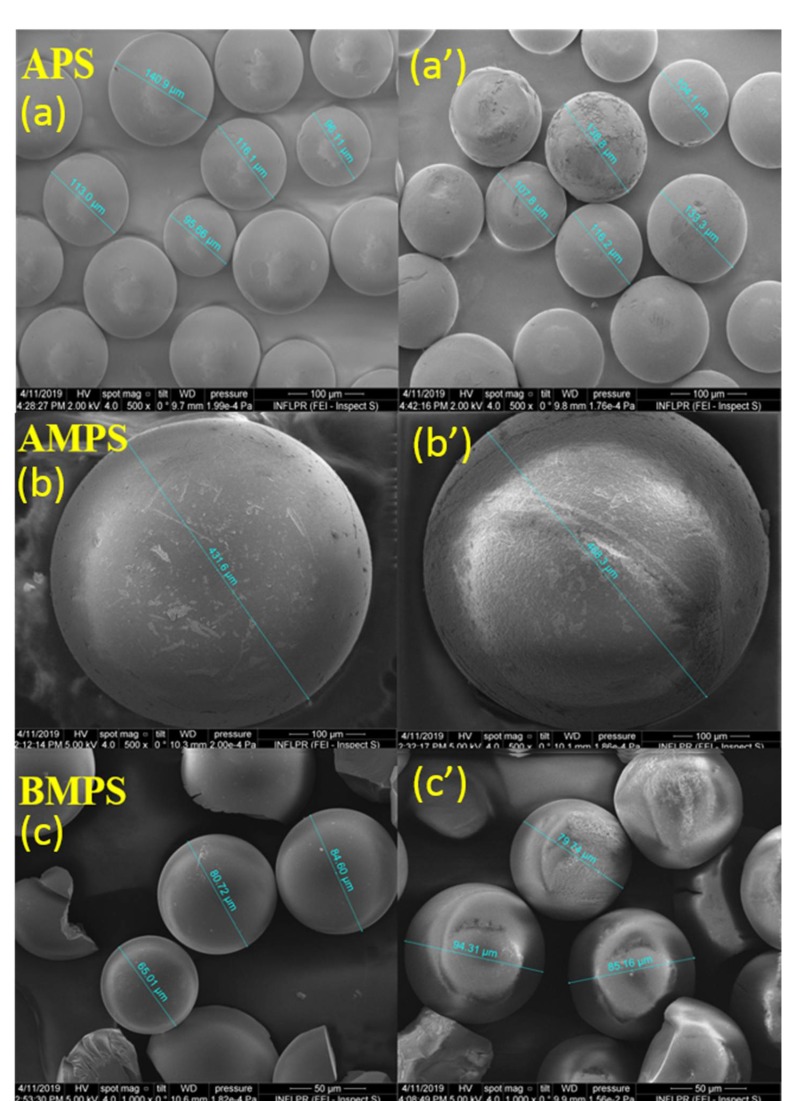
SEM microphotographs of the solid catalysts before (**a**), (**b**), (**c**) and after four cycles of pullulan oxidation reaction (**a’**), (**b’**), (**c’**).

SEM analyses of the three solid catalysts were performed before and after the four cycles of pullulan oxidation in order to determine the morphological changes appearing during exposure of catalysts to the highly alkaline reaction medium. As can be seen from Figure 6, the overall integrity of the catalysts was maintained even after four reaction cycles. All of the catalysts show a spherical shape, with sizes varying from ~100 to 150 μm (sample APS), ~500 μm (sample AMPS), and ~65−85 μm (sample BMPS), respectively.

After their utilization in four reaction cycles, their shapes do not undergo any visible changes, the size remaining unaltered. A slight modification can be observed in the cases of AMPS and BMPS samples, their spherical shape becoming swollen in appearance, but without affecting their catalytic performance or destroying the initial shape and morphology.

## 4. Conclusions

For the first time, we propose the use of recyclable polymer-supported *N*-hydroxyphthalimide (NHPI) catalysts for the selective oxidation of primary OH groups in pullulan. The proposed oxidation protocol is simple, efficient, and occurs under mild reaction conditions. The catalyst could be used for at least four reaction cycles. The polyelectrolyte titrations by means of particle charge detector, performed on oxidized samples, reveal different values for electrokinetic charge depending on the catalyst type and its number of uses, with the highest electrokinetic charge being found in the case of the sample oxidized with NHPI immobilized on chloromethyl polystyrene via ester bonds, BMPS-recycle 3 (EC~35%). The morphological integrity of the solid catalysts, as revealed by SEM analyses, is maintained for prolonged utilization times. These catalysts could represent cheaper and valuable alternatives to those expensive ones which exist today, i.e., TEMPO.

## Figures and Tables

**Table 1:** Types of catalysts used in reactions.

Catalyst	Immobilized by	Crosslinking Degree of Resin (% DVB)	NHPI Loading, Our Work, (mmol NHPI/g)	NHPI Loading, Ref. [20] (mmol NHPI/g)
APS-NHCO-NHPI-1 (APS)	Amide bond	2.0	1.6	1.12
AMPS-OCO-NHPI-1 (AMPS)	Ester bond	5.5	2.1	1.65
BMPS-OCO-NHPI-2 (BMPS)	Ester bond	2.0	0.9	0.9

**Table 2:** Electrokinetic charge (EC) and zeta potential (ζ) for pullulan samples oxidized in the presence of the NHPI-supported catalysts.

Pullulan (g)	Catalyst			NaBr (mM/g)	NaOCl (mM/g)	EC (%)	ζ (mV)
	Type	Recycle	Amount (g)				
1	APS-NHCO-NHPI-1 (APS)	0	0.8	1	10	21 ± 0.4	−19.7 ± 0.6
		1	0.75	0.94		26 ± 0.2	−19.5 ± 0.4
		2	0.6	0.76		27 ± 0.3	−18.8 ± 0.2
		3	0.5	0.63		27 ± 0.6	−22.1 ± 0.8
1	AMPS-OCO-NHPI-1 (AMPS)	0	0.8	1	10	17 ± 0.1	−18.6 ± 0.2
		1	0.75	0.94		21 ± 0.3	−17.6 ± 0.6
		2	0.7	0.88		13 ± 0.9	−16.2 ± 0.4
		3	0.6	0.86		16 ± 0.6	−16.6 ± 0.2
1	BMPS-OCO-NHPI-2 (BMPS)	0	0.8	1	10	17 ± 0.5	−24.7 ± 0.2
		1	0.75	0.96		18 ± 0.3	−21.8 ± 0.2
		2	0.65	0.85		27 ± 0.4	−24.9 ± 0.5
		3	0.5	0.65		35 ± 1.5	−27.3 ± 0.6

## Supplementary Materials

The following are available online at https://www.mdpi.com/1996-1944/12/21/3585/s1, Figure S1: The ^13^C-NMR spectra of PI – APS, Figure S2: The ^13^C-NMR spectra of PII – APS, Figure S3: The ^13^C-NMR spectra of PI – AMPS, Figure S4: The ^13^C-NMR spectra of PII – AMPS, Figure S5: The ^13^C-NMR spectra of PI – BMPS, Figure S6. The ^13^C-NMR spectra of PII – BMPS.
