# Supplementary material for: Recyclable Polymer-Supported N-Hydroxyphthalimide Catalysts for Selective Oxidation of Pullulan
Source: Materials (Basel). 2019 Oct 31;12(21):3585. doi: 10.3390/ma12213585 (PMC6862700; doi:10.3390/ma12213585)
Supplement: Supplementary file 1 [file materials-12-03585-s001.pdf]

materials

Article

# Recyclable Polymer-Supported *N*-Hydroxyphthalimide Catalysts for Selective Oxidation of Pullulan

**Madalina Elena Culica [1], Kornela Kasperczyk [2], Raluca Ioana Baron [1], Gabriela Biliuta [1], Ana Maria Macsim [1], Andrada Lazea-Stoyanova [3], Beata Orlinska [2] and Sergiu Coseri [1,*]**

1 "Petru Poni" Institute of Macromolecular Chemistry of Romanian Academy, 41 A, Gr. Ghica Voda Alley, Iasi 700487, Romania; culica.madalina@icmpp.ro (M.E.C.); baron.raluca@icmpp.ro (R.I.B.); biliuta.gabriela@icmpp.ro (G.B.); ana.iurascu@icmpp.ro (A.M.M.)

2 Department of Chemical Organic Technology and Petrochemistry, Silesian University of Technology, Krzywoustego 4, Gliwice 44 100, Poland; kornela.kasperczyk@gmail.com (K.K.); Beata.Orlinska@polsl.pl (B.O.)

3 National Institute for Lasers, Plasma and Radiation Physics, 409 Atomistilor Street, Magurele 77125, Romania; andrada@infim.ro

* Correspondence: coseris@icmpp.ro ; Tel.: +40–232–217–454; Fax: +40–232–2112–99

Received: 26 August 2019; Accepted: 30 October 2019; Published: date

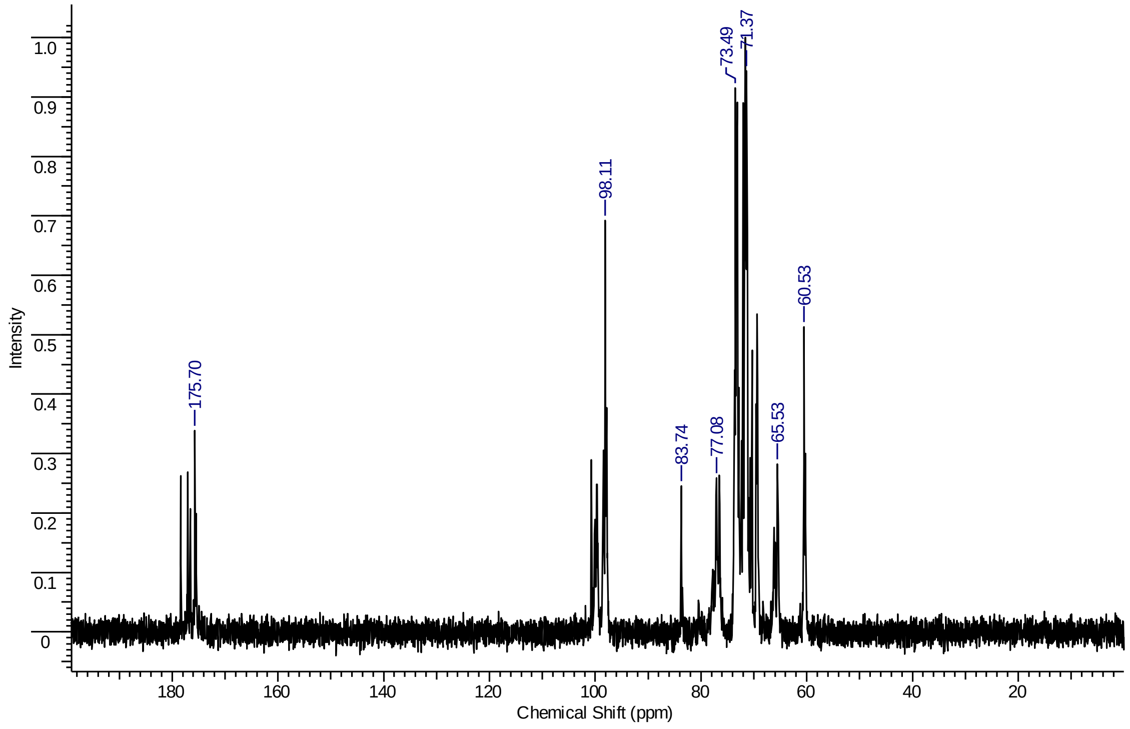


**Figure S1.** The $^{13}$C-NMR spectra of PI - APS.

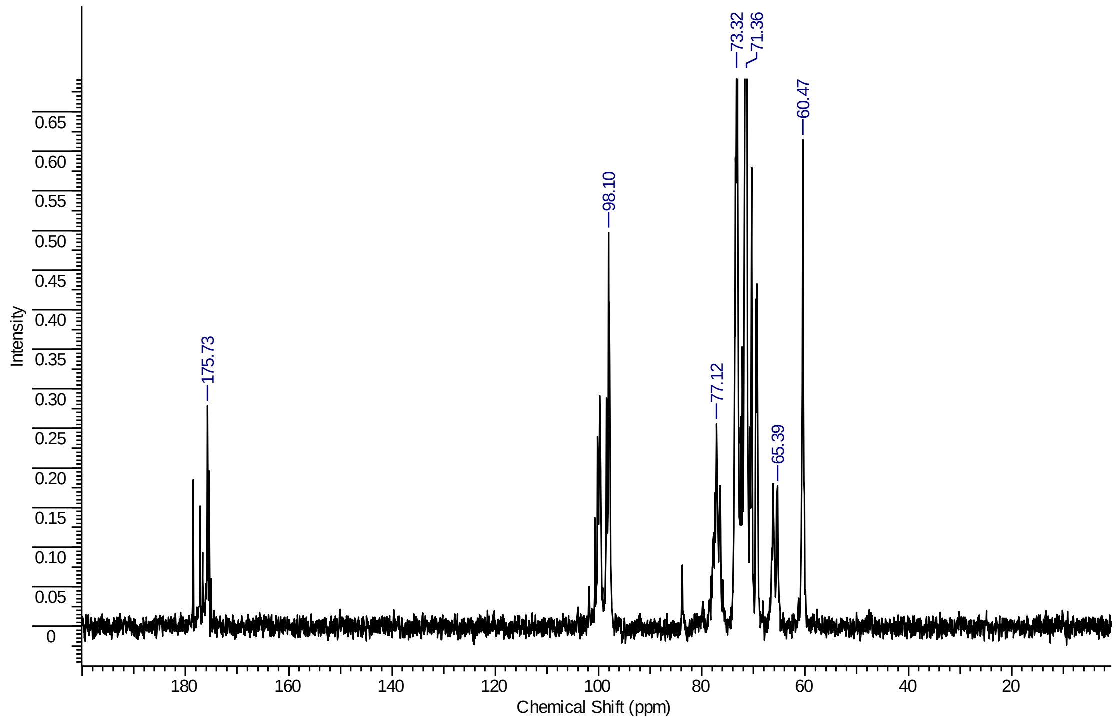


**Figure S2.** The $^{13}$C-NMR spectra of PII - APS.

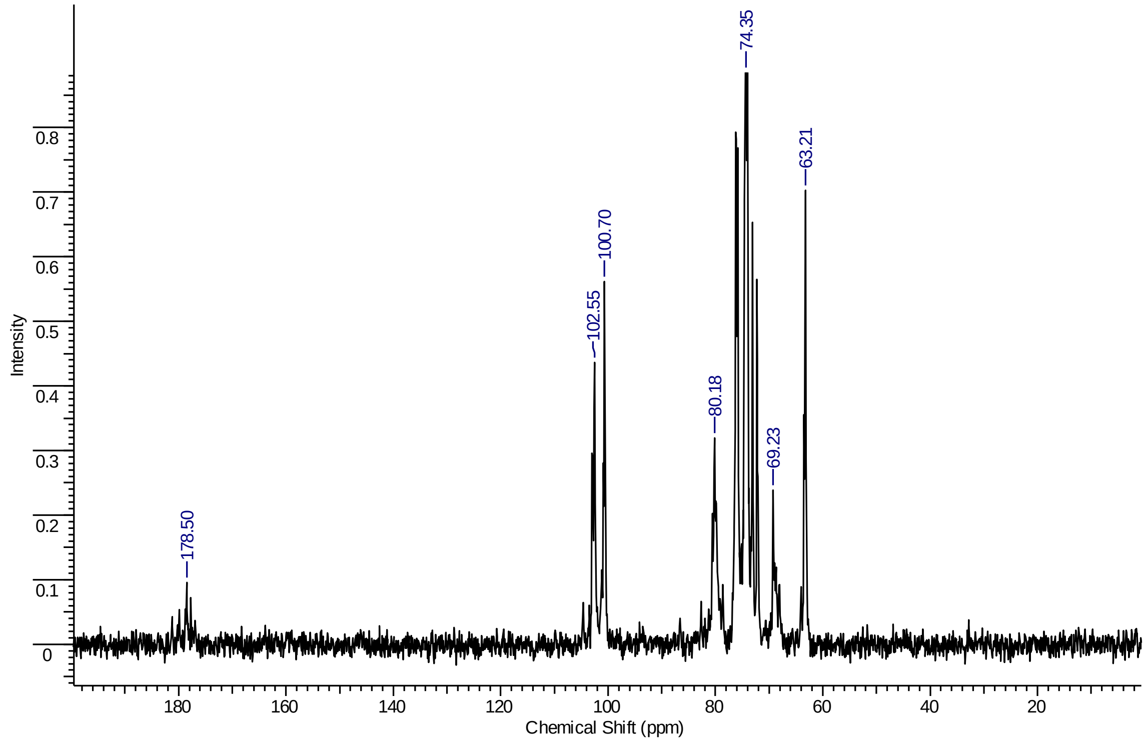


**Figure S3.** The $^{13}$C-NMR spectra of PI - AMPS.

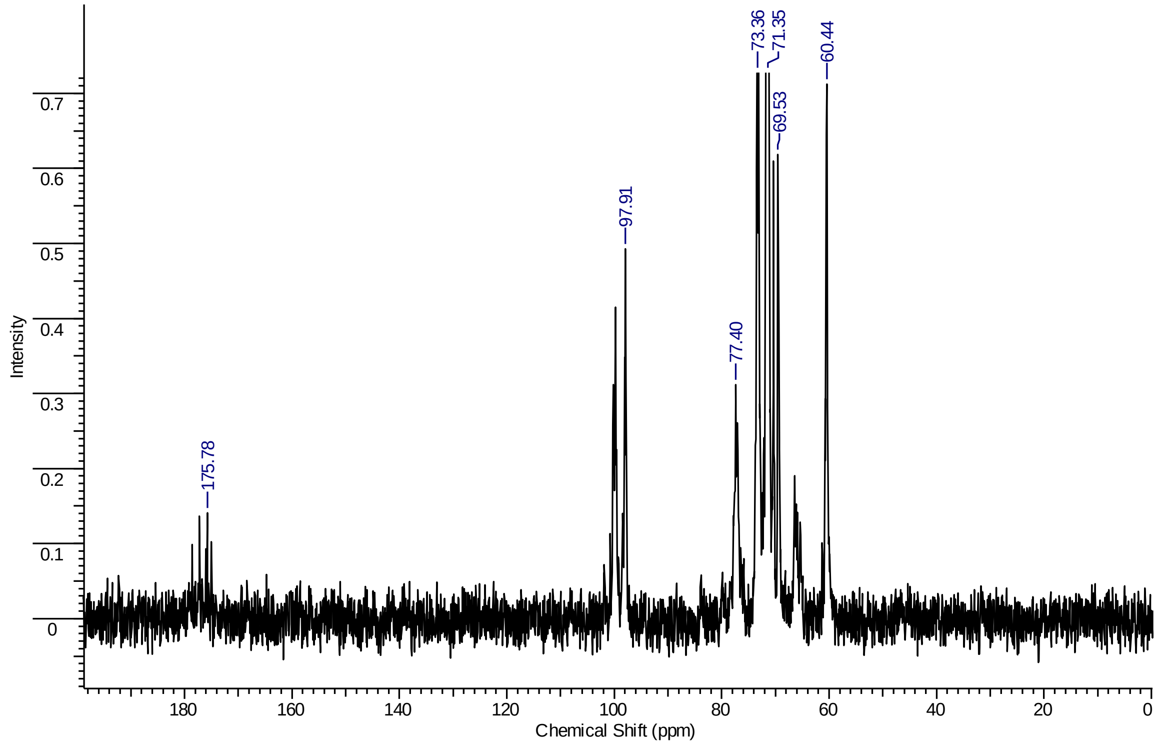


**Figure S4.** The $^{13}C$-NMR spectra of PII - AMPS.

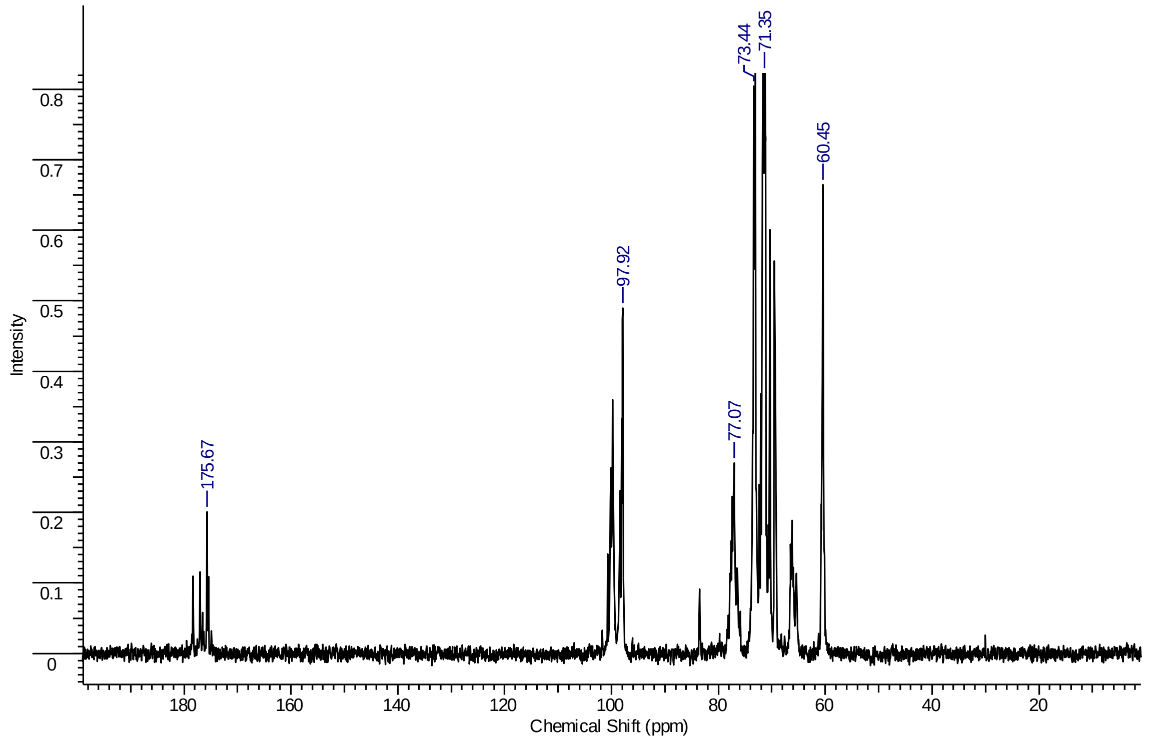


**Figure S5.** The $^{13}C$-NMR spectra of PI - BMPS.

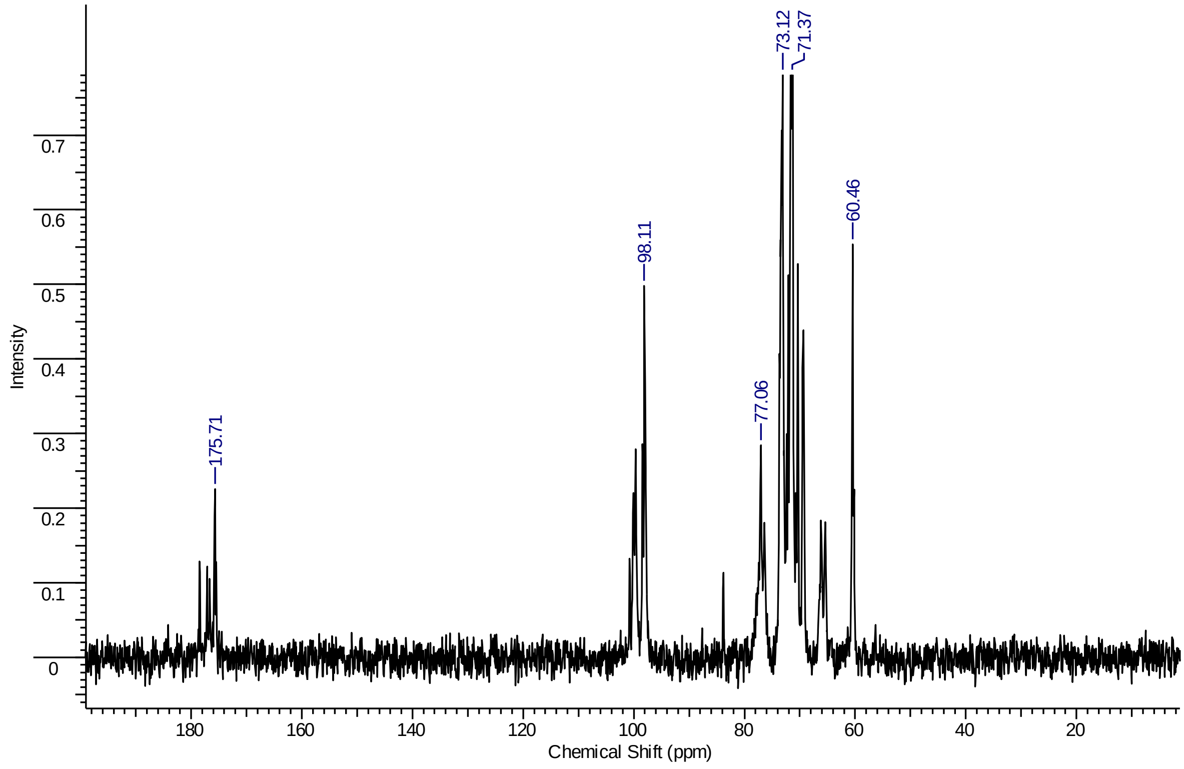


**Figure S6.** The $^{13}C$-NMR spectra of PII - BMPS.
